# Sentinel Lymph Node Mapping in High-Grade Endometrial Cancer

**DOI:** 10.3390/curroncol29020096

**Published:** 2022-02-14

**Authors:** Lina Salman, Maria C. Cusimano, Zibi Marchocki, Sarah E. Ferguson

**Affiliations:** 1 Division of Gynecologic Oncology, Department of Obstetrics & Gynecology, University of Toronto, 610 University Ave, Toronto, ON M5G2M9, Canada; lina.salman@uhn.ca (L.S.); mcusimano@qmed.ca (M.C.C.); zbigniew.marchocki@uhn.ca (Z.M.); 2 Division of Gynecologic Oncology, Princess Margaret Cancer Centre/Sinai Health Systems, Toronto, ON M5G2M9, Canada

**Keywords:** endometrial cancer, high-grade, sentinel lymph node

## Abstract

Sentinel lymph node (SLN) mapping is becoming an acceptable alternative to full lymphadenectomy for evaluating lymphatic spread in clinical stage I endometrial cancer (EC). While the assessment of pelvic and para-aortic lymph nodes is part of the surgical staging of EC, there is a long-standing debate over the therapeutic value of full lymphadenectomy in this setting. Although lymphadenectomy offers critical information on lymphatic spread and prognosis, most patients will not derive oncologic benefit from this procedure as the majority of patients do not have lymph node involvement. SLN mapping offers prognostic information while simultaneously avoiding the morbidity associated with an extensive and often unnecessary lymphadenectomy. A key factor in the decision making when planning for EC surgery is the histologic subtype. Since the risk of lymphatic spread is less than 5% in low-grade EC, these patients might not benefit from lymph node assessment. Nonetheless, in high-grade EC, the risk for lymph node metastases is much higher (20–30%); therefore, it is crucial to determine the spread of disease both for determining prognosis and for tailoring the appropriate adjuvant treatment. Studies on the accuracy of SLN mapping in high-grade EC have shown a detection rate of over 90%. The available evidence supports adopting the SLN approach as an accurate method for surgical staging. However, there is a paucity of prospective data on the long-term oncologic outcome for patients undergoing SLN mapping in high-grade EC, and more trials are warranted to answer this question.

## 1. Introduction

Sentinel lymph node (SLN) mapping is a minimally invasive approach developed to identify occult metastases in normal-appearing lymph nodes, while avoiding complete pelvic lymph node dissection [1]. This technique is derived from the presumption that the lymphatic drainage of the primary tumor proceeds in a predictable stepwise fashion from the most proximal lymph node to the tumor site (i.e., SLN) to more distal lymph nodes (upper echelon). If the SLN is negative, then the remaining lymph nodes in that lymphatic chain should theoretically also be negative [2]. SLN mapping is associated with reduced surgical morbidity and postoperative lymphedema compared to complete lymphadenectomy [3], but has similar, if not improved, diagnostic accuracy for nodal metastases [2]. Moreover, since most patients will not have any lymph node metastases at the time of surgery, full lymph node dissection is unnecessary and can be avoided with SLN mapping [4,5].

The SLN technique was first described in 1960 by Gould for the management of parotid cancer [6], and later applied by Cabanas to the treatment of penile carcinoma [7]. The use of SLN mapping ultimately expanded to other solid tumors, including breast [8], melanoma [9], and vulvar cancers [10]. SLN mapping is now the standard of care for surgical staging of patients with early-stage breast cancer and melanoma given its excellent performance characteristics and safety profile. In randomized controlled trials (RCTs) of breast cancer patients, overall survival is similar between those undergoing SLN biopsy and those undergoing axillary lymph node dissection, regardless of lymph node status [11,12]. In melanoma patients with positive SLN, there was no difference in cancer-specific survival in patients randomized to immediate regional lymph node dissection versus no further surgery [13]. In addition, patients who had only SLN mapping had a significantly decreased rate of lymphedema (6.3%) compared to regional lymph node dissection (24%).

SLN mapping has been evaluated and incorporated into standard practice for multiple gynecologic malignancies including vulvar, cervical, and endometrial cancer [14,15,16]. The first gynecologic disease site to incorporate the SLN concept was vulvar cancer. In this setting, SLN mapping has a sensitivity of 87% (66–97%) per patient, and an NPV of 100% (95–100%) [16,17]. The GROINSS-V was a prospective cohort study that evaluated the oncologic outcomes of patients with vulvar cancer who had a negative SLN and in whom inguinofemoral lymphadenectomy was omitted [5]. Of the 259 patients included in the analysis, groin recurrence rate was 2.3% and the three-year survival rate was 97%. The rates of lymphedema were also far lower in patients undergoing SLN biopsy compared to lymphadenectomy (1.9% vs. 25%). These findings have led to the practice of omitting regional lymph node dissection in vulvar cancer when SLNs are negative. Recently, the GROINSS-V II prospective cohort study evaluated whether the use of inguinofemoral radiotherapy (50 Gy) could replace complete inguinofemoral lymph node dissection in patients with vulvar cancer (<4 cm) who have a positive SLN [18]. Due to a high rate of groin recurrence in patients with macrometastases (>2 mm), the protocol was amended to include complete inguinofemoral lymph node dissection in those with macrometastases only. This study ultimately demonstrated an acceptable low rate of groin recurrence (1.6%) in patients with SLN micrometastases who received radiotherapy only. Nevertheless, the rate of groin recurrence was high at 22% for those who received radiotherapy only for macrometastases, compared to 6.9% in those who had complete inguinofemoral lymph node dissection. These findings highlight the need to evaluate SLN mapping for each cancer type and identify the patients with suitable clinicopathologic tumor characteristics for which the SLN technique can be performed with high accuracy and safety.

SLN mapping was first performed for endometrial cancer (EC) in 1996 by Burk et al. [14]. This was a pilot study of fifteen patients with high-grade EC and was the first step towards a paradigm shift in surgical staging for EC. Since then, the techniques for SLN mapping have evolved. Multiple international guidelines recommend that SLN mapping replace systematic pelvic and para-aortic lymph node dissection in patients with low-grade EC, and be considered as a reasonable alternative for high-grade subtypes of EC [19,20,21]. The literature on SLN in EC is diverse, addressing questions of feasibility and accuracy, across different injection techniques and dyes, in both prospective and retrospective manners. Many pivotal prospective studies evaluating SLN technique in EC included patients with different histologic subtypes and predominantly low-grade EC [22,23]. Questions have been raised about the accuracy of this technique in patients with high-grade histologic subtypes due to the greater risk for lymph node metastases and concerns about alternative lymphatic drainage resulting in isolated para-aortic lymph node involvement [24,25,26]. In this review, we will discuss the role of SLN mapping in EC, focusing on patients with high-grade histology [27].

## 2. Lymph Node Assessment in Endometrial Cancer

EC is the most common gynecologic malignancy and the fourth most common cancer in women in North America, with a cumulative risk of 2.5% to 75 years of age [28]. The incidence of EC has increased by 1% per year over the last 15 years [29,30]. Lymph node involvement is the most important prognostic factor in EC [31]. The Gynecologic Oncology Group (GOG-33) prospective surgical staging study highlighted the significant rate of occult lymph node metastases (22%) in clinical stage 1 EC and the importance of surgical staging and pathologic evaluation of regional lymph nodes in EC to individualize adjuvant therapy [32]. Identifying lymph node metastases not only provides prognostic information, but also helps guide adjuvant treatment, as these patients with node-positive disease will benefit [33,34]. This led to a shift from clinical to surgical staging as recommended by the International Federation of Gynaecology and Obstetrics (FIGO) in 1988 [35].

## 3. Is Lymphadenectomy Therapeutic?

Two large RCTs addressed the therapeutic role of lymphadenectomy versus no lymphadenectomy in clinical stage I EC [4,36]. Both studies failed to show an overall survival advantage for patients undergoing full lymphadenectomy. One of the major limitations of these studies is that most of the recruited patients had low-grade EC, which is associated with a low rate of nodal involvement and mortality; as such, these studies may not have had the statistical power to detect a therapeutic benefit associated with lymphadenectomy [36,37]. The ASTEC trial was additionally criticized for high rates of adjuvant radiation therapy regardless of lymph node status, which could have influenced the results [4]. The results from these RCTs have led to a conceptual shift regarding the role of lymphadenectomy from therapeutic procedures to diagnostic procedures for individuals with clinical stage I disease in order to identify occult advanced stage disease and tailor adjuvant therapy. This has paved the way to incorporating the SLN concept in surgical staging of EC, an approach that accurately identifies lymph node metastases while sparing the detrimental side effects of full lymphadenectomy.

## 4. Sentinel Lymph Node Mapping in Endometrial Cancer

### 4.1. Injection Sites and Tracers

**Figure 1:**
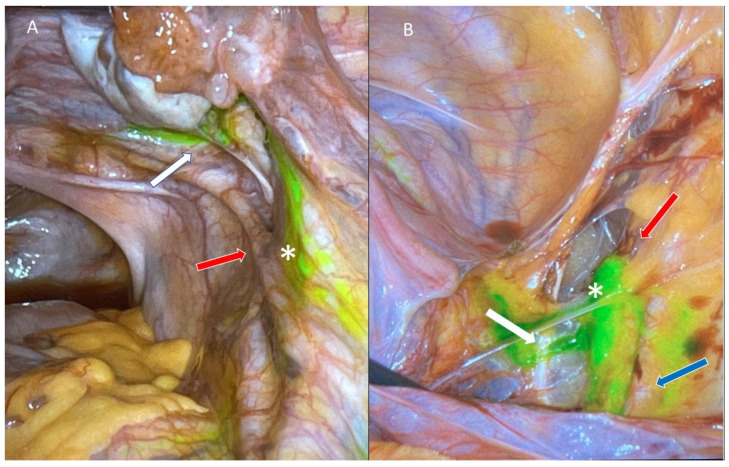
Mapping of a sentinel lymph node using cervical injection of indocyanine green. (**A**) Parametrial lymphatic chains (white arrow) leading to the right external iliac sentinel lymph node (*), while identifying the adjacent structures such as the right ureter (red arrow). (**B**) Right external iliac sentinel lymph node (*) clearly identified after exposing the retroperitoneal space. Important vessels identified in the retroperitoneal space: superior vesicle artery (white arrow), right external iliac vein (red arrow), and right external iliac artery (blue arrow).

Since the initial description of the SLN mapping in EC, many different protocols for SLN mapping have been investigated, including different injection sites and tracers [38,39]. Cervical, sub-serosal, myometrial, and hysteroscopically guided tumor injection have all been evaluated [21]. The injection of tracer into the cervix, superficially and deep at two locations (three and nine o’clock), is the most common site of injection [2]. Although cervical injection has been associated with lower rates of para-aortic SLN detection compared to uterine injection (6.7% vs. 26.8%, respectively), it has become the most acceptable approach due to its high SLN detection rate overall [40] (Figure 1).

**Figure 2:**
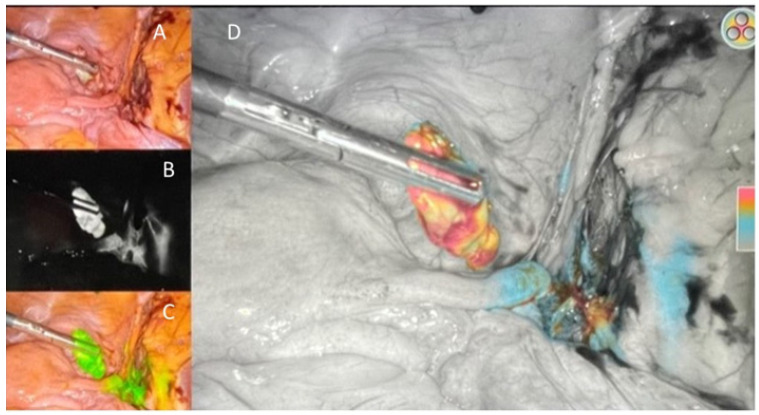
Sentinel lymph node in different views using laparoscopic near-infrared technologies. (**A**) High definition (HD)—white light; (**B**) spy-fluorescence mode; (**C**) pinpoint fluorescence; (**D**) color segmental fluorescence.

Multiple tracers have been used for SLN mapping in EC, including blue dye, technetium-99 (Tc-99), and indocyanine green (ICG). The combination of Tc-99 and blue dye has an average detection rate of 86.3% (80.7–91.9%) [41]. However, this tracer requires collaboration with a nuclear medicine unit and preoperative imaging, which increases the cost and requires a painful preoperative injection for patients [42]. One prospective study compared blue dye alone to ICG alone and found a significantly higher detection rate with ICG (90.9% vs. 64.4%) [38]. The COMBITEC study compared the combination of ICG and Tc-99 to ICG alone and found similar bilateral pelvic and para-aortic mapping rates, concluding that there is no benefit to addition of Tc-99 over ICG alone [43]. Systematic reviews and meta-analyses of over 40 published trials comparing different tracers report that ICG has the highest pooled detection rate (90–94%) [44,45,46]. Currently, the use of ICG dye with near-infrared fluorescent imaging is recommended by the Society of Gynecologic Oncology due to its technical simplicity, high detection rate, and reliability [21] (Figure 2).

### 4.2. Sentinel Lymph Node Algorithm and Ultra-Staging

A group at Memorial Sloan-Kettering Cancer Center (MSKCC) has developed an algorithm for SLN mapping that is widely accepted and incorporated into practice [20,47,48]. In short, the procedure starts by injecting dye into the cervix at the time of examination under anesthesia. The first step in the surgery is the inspection of peritoneal and serosal surfaces and the collection of pelvic washings. On each hemi-pelvis, mapped SLN and suspicious nodes (regardless of mapping) are resected. If no SLN is identified on a hemi-pelvis, a side-specific pelvic, common iliac, and interiliac lymph node dissection is performed [47]. While para-aortic lymphadenectomy is performed as per the surgeon’s discretion, many surgeons will perform this step in patients with high-grade EC in addition to pelvic SLN mapping. The pathologic evaluation of SLN consists of an initial examination with hematoxylin and eosin (H&E), followed by ultra-staging if H&E is negative [47]. Ultra-staging refers to serial sectioning of the lymph node for both H&E staining and immunohistochemistry for cytokeratin. It is performed in cases where SLNs are negative for metastases in routine H&E staining. This technique improves the identification of nodal metastases, especially for low-volume disease that would have been missed on traditional pathologic assessment [49,50]. The use of this algorithm and ultra-staging in clinical stage I EC results in the identification of node-positive disease and upstaging in approximately 36% of cases [26,49].

### 4.3. Benefits of Sentinel Lymph Node Mapping

The advantages of SLN mapping lie in the higher rate of identifying node-positive disease and lower morbidity compared to complete lymphadenectomy [50,51]. SLN dissection is less invasive than a complete pelvic and para-aortic lymphadenectomy, resulting in lower rates of intra-operative complications and post-operative lymphedema [51]. Previous studies have shown that use of SLN mapping for EC staging was associated with decreased blood loss, reduced surgery time by almost 90 min, and a decreased risk of vascular and nerve injuries compared to full pelvic and para-aortic lymphadenectomy [51,52,53]. Moreover, the risk for lower extremity lymphedema is significantly reduced in SLN procedures compared to lymphadenectomy, from 40% to 27%, as self-reported by patients [54]. All of these advantages make SLN mapping the preferred approach for surgical staging in EC.

### 4.4. Detection Rate and Sensitivity

Several important parameters are evaluated when assessing the accuracy of the SLN technique. The detection rate for SLN mapping is the proportion of cases in which a SLN is identified. The acceptable detection rate for SLN mapping is considered to be >90% [2,55], and this depends both on the surgical technique used and the surgeon’s experience [56,57]. The sensitivity of SLN mapping is the proportion of patients with nodal metastases that were correctly identified by the procedure and is reported in EC to be 91–98% [25,26,58,59].

The accuracy of SLN biopsy was initially evaluated in retrospective studies, using different tracers and injection sites, with detection rates of 45–87% [60,61,62]. This led to several prospective trials that aimed to evaluate the sensitivity of SLN mapping compared to complete lymphadenectomy as the reference standard [22,23,25,26,63]. The SENTI-ENDO trial was one of the first prospective studies to evaluate the accuracy of SLN in EC; the recruited patients had a SLN assessment followed by pelvic lymphadenectomy as the reference standard [22]. Using a cervical injection of Tc-99 combined with blue dye, they reported a detection rate of 89%, a NPV of 97%, and a sensitivity of 84% per patient. This study contributed greatly to our understanding of the accuracy of the SLN mapping technique, but it did not use ICG as the tracer, and it did not evaluate the sensitivity of the SLN algorithm, which would have likely increased the sensitivity per patient. In addition, 87% of the patients had low-grade EC, making it difficult to generalize the results to the high-grade population.

The FIRES trial was a large multicenter prospective trial aiming to evaluate the sensitivity and NPV of SLN mapping in clinical stage 1 EC using ICG compared to complete lymphadenectomy (pelvic with or without para-aortic lymphadenectomy) [23]. This study reported a detection rate of 86%, a sensitivity of 97.2%, and an NPV of 99.6%. The FIRES trial provided further evidence supporting the adoption of the SLN approach in EC staging, but only 28% of the cohort had high-grade histology.

Several prospective studies have evaluated the role of SLN mapping in patients with high-risk EC [24,25]. A prospective study conducted by the group at MD Anderson Cancer Center [24] examined SLN mapping in patients with either a high-grade histologic subtype, cervical involvement, or FIGO grade 1/2 with suspected deep myometrial invasion on imaging. All patients underwent SLN mapping followed by pelvic and para-aortic lymphadenectomy. Of the 101 cases that were ultimately evaluated, the detection rate per patient was 89%, and the bilateral detection rate was 58%. SLN biopsy alone accurately identified 95% of patients with positive lymph nodes. Only one patient had bilateral negative SLN and positive non-sentinel lymph nodes upon final pathology. These findings suggested that SLN mapping remained accurate in patients at higher risk of nodal involvement, including those with high-grade histology who comprised just over 50% of the cohort.

The Pelvic Sentinel lymph node detection in High-Risk Endometrial Cancer (SHREC) trial was a Swedish prospective study designed to evaluate the diagnostic accuracy of pelvic SLN in high-risk EC [25]. Patients with clinical stage I–II EC with at least one the following preoperative high-risk criteria were enrolled: high-grade histology, deep myometrial invasion, cervical stromal invasion, or non-diploid cytometry [25]. Using cervical injection of ICG, SLNs were identified and dissected followed by pelvic and infrarenal para-aortic lymphadenectomy. The investigators reported a bilateral mapping rate of 95%, a sensitivity of 98%, and a NPV of 99.5%. Although encouraging, the accuracy of the SLN algorithm was not assessed for the specific subgroup of patients with high-grade histology (49% of those enrolled).

Most prospective studies evaluating SLN mapping included patients with clinical stage I disease [25]; however, the trends and outcomes of SLN mapping for stage II EC were recently evaluated in a population-based study [64]. Data on over 6000 patients with stage II EC showed that the utilization of SLN mapping increased annually by 45% between 2010 and 2018, with a comparable three-year overall survival rate between patients undergoing SLN mapping and patients with lymphadenectomy. The validity of SLN mapping in stage II EC should be evaluated in further prospective studies.

## 5. Accuracy of Sentinel Lymph Node Mapping in High-Grade Endometrial Cancer

High-grade EC refers to any histology other than grade 1–2 endometroid EC, including: grade 3 endometrioid carcinoma, clear cell carcinoma, serous carcinoma, carcinosarcoma, and undifferentiated and mixed histology [65,66]. This subgroup of EC is more aggressive as it tends to disseminate outside the uterus earlier in the course of disease [27]. About 20–40% of patients with high-grade histology will have nodal metastases upon presentation, compared to less than 5% of those with low-grade histology [26,67,68,69]. While controversies exist over the value of assessing lymphatic spread in low-grade histology, it is essential to determine lymph node status in patients with high-grade histology to deliver the proper adjuvant treatment [33,34].

### 5.1. Sentinel Lymph Node in High-Grade Histology

The surgical management for clinical early-stage high-grade EC is similar to that for low-grade EC and consists of surgical staging with lymph node assessment. Although the previously mentioned prospective studies included patients with high-grade histologic subtypes, this population comprised less than 50% of each cohort, and the role of SLN mapping in this specific subgroup was not directly evaluated [22,23,24,25]. Retrospective studies aiming to evaluate the utility of SLNs in patients with high-grade EC [70,71] either had a small number of patients [70] or were not able to determine sensitivity and NPV since a reference complete lymph node dissection was not performed [71].

The SENTOR study aimed to evaluate the diagnostic accuracy and performance characteristics of SLN mapping in patients with predominantly high-grade (80% of the cohort) clinical stage 1 EC [26]. While previous studies focused on high-risk EC including any of the following FIGO grade 1 or 2 endometrioid histology with ≥50 myometrial invasion or cervical stromal invasion [24,25], the SENTOR study was the first prospective trial that excluded low-grade EC. A total of 156 patients were enrolled and underwent the SLN algorithm proposed by MSKCC followed by complete pelvic lymphadenectomy (grade 2 endometrioid) and pelvic plus para-aortic lymphadenectomy (all high-grade histotypes). The SLN detection rates were 77.6% bilaterally and 97.4% per patient. Of the entire cohort, 17% had nodal metastasis. Only one patient with positive nodal metastasis was not identified by the SLN algorithm, yielding a sensitivity of 96% and a NPV of 99%. The results of this study were consistent with those of the previous literature and highlighted the high diagnostic accuracy of SLN in high-grade EC, cementing SLN mapping as an acceptable surgical staging approach for all clinical stage I EC.

The diagnostic accuracy of SLN mapping using ICG in high-grade EC was more recently evaluated in a systematic review and meta-analysis [72]. All the prospective studies of patients with clinical stage 1 high-grade EC undergoing SLN mapping with cervical injection of IGC and at least bilateral pelvic LND as the reference standard were identified. A total of 9 studies published prior to January 2021 and providing data on 429 patients were included in the meta-analysis. This demonstrated an overall SLN detection rate of 91% per patient and a bilateral detection rate of 64% (50–81%). The SLN sensitivity and NPV per patient were 92% and 97%, respectively. This study was unable to assess the performance of the SLN algorithm as this was not the primary objective of most of the included studies. Regardless, the findings provided additional evidence supporting the incorporation of SLN mapping for patients with high-grade EC.

### 5.2. Isolated Para-Aortic Lymph Node Metastases

Lymph node metastases in EC are commonly confined to the pelvic lymph nodes. Even when para-aortic lymph nodes are found to be positive, the pattern of spread is usually systematic and involves the pelvic lymph nodes before reaching the para-aortic through afferent lymphatic chains. Isolated para-aortic lymph node metastases with negative pelvic nodes, although rare (<2%), can occur through ovarian vessels or through presacral lymph node channels, and have been associated with lymphovascular space invasion and high-grade histologic subtypes [73,74]. This has led to concerns over missing isolated para-aortic nodal disease (stage IIIC2 disease) and would have a significant impact on prognosis and adjuvant therapy for these patients [35]. Therefore, some have advocated for SLN mapping with additional para-aortic lymphadenectomy for individuals with high-grade EC [20].

The patterns of lymphatic spread and the safety of the SLN algorithm in patients with high-grade histology were reported by the SENTOR study. Overall, 4% of the patients had SLN mapped in the para-aortic region, but all of these lymph nodes were negative for metastases. Seven patients (6%) had both pelvic and para-aortic metastases, and only one patient (0.8%) had isolated para-aortic metastases identified using the SLN algorithm (complete pelvic and para-aortic lymphadenectomy) due to failed mapping on that side [26,47]. Similar to these findings, only three patients (<1%) had SLN identified in the para-aortic region in the FIRES study, and only two (0.56%) of these patients had isolated para-aortic metastases [23]. One additional patient was found to have isolated para-aortic lymph node metastases upon full lymphadenectomy, after failed SLN mapping on that side. The low rate of isolated para-aortic lymph node metastases in these studies support the implementation of the SLN algorithm in high-grade EC and abandoning routine para-aortic lymphadenectomy.

### 5.3. Oncologic Outcome following Sentinel Lymph Node Mapping

SLN mapping can accurately detect lymphatic spread, but whether recurrence-free and overall survival are compromised with this procedure compared to complete lymphadenectomy remains unclear [26,72]. There are no prospective data comparing the oncologic outcomes of SLN mapping compared to lymphadenectomy, but retrospective studies have failed to show any significant difference in survival in patients with confirmed nodal metastases undergoing SLN mapping versus traditional lymphadenectomy [75,76]. One could argue that removing only the mapped SLNs might result in worse oncologic outcomes since part of the lymphatic channels that remains in situ or a non-SLN could be involved in metastatic disease. However, the majority of the patients diagnosed by nodal metastasis through SLN mapping will eventually receive adjuvant treatment, which will treat occult metastases [34]. In addition, the resected SLN is the only site of disease in over 50% of patients with high-grade EC, and this SLN often has low-volume metastases [23,26]. A recent retrospective study comparing outcomes in patients undergoing lymphadenectomy alone with patients undergoing SLN followed by complete lymphadenectomy found that the addition of SLN resulted in improved overall and progression-free survival and lower rates of pelvic recurrence [77]. These findings suggest that SLN improved diagnostic accuracy and provided additional details for decisions on adjuvant treatment that could improve outcomes.

## 6. Sentinel Lymph Mapping in the Era of Molecular Classification

Moving beyond the current type I (low-grade) and II (high-grade) histopathologic classification, The Cancer Genome Atlas (TCGA) Research Network identified four molecular subgroups of EC based on mutational load and somatic copy number alterations (SCNA) that have distinct clinical outcomes and potential for novel targeted therapies [78]. The four groups include: (*1*) *ultra-mutated*, characterized by an excellent prognosis and high mutation rate due to mutations of the exonuclease domain of *POLE*; (*2*) *microsatellite instability high-hyper-mutated*, an intermediate prognosis subtype; (*3*) *SCNA-low*, an intermediate prognosis subtype characterized by low mutational burden; and (*4*) *SCNA-high*, a poor prognosis subtype characterized by low mutation burden, serous histology, *p53* mutations (*p53*mut), and significantly worse survival compared to other subgroups. These four groups can be determined using surrogate markers in paraffin-embedded tissues, with analogous groups as follows: *POLE* ultra-mutated, mismatch repair deficient (MMRd), no specific molecular profile, and *p53*mut [79].

PORTEC 3, an RCT evaluating the addition of chemotherapy to radiation for high-risk EC stage I to III, found an overall survival benefit with the addition of chemotherapy to all patients with serous EC (regardless of stage) [34,80]. Most importantly, they found that *p53*mut EC, regardless of histologic subtype, was the only TCGA molecular group that benefited from adjuvant chemotherapy with radiation compared to radiation alone [79]. In addition, there is growing evidence that the MMRd subgroup does not benefit from adjuvant chemotherapy and that the *POLE* subgroup, which is often associated with high-grade EC, has an excellent prognosis, and de-escalation of treatment may be appropriate [79]. The impact of individualized adjuvant treatment based on molecular profiles, compared to standard adjuvant treatment, is currently being assessed in the on-going PORTEC-4a study [81].

A large majority of patients with high-grade EC have tumors that are *p53*mut, and this therefore brings into question the role of SLN mapping in these patients. If the *p53*mut subgroup benefits from adjuvant chemotherapy regardless of stage of disease, would there still be any diagnostic or therapeutic value in SLN mapping beyond accurately determining stage and prognosis? On the contrary, SLN mapping in cases with *p53* wildtype might be of greater importance. Identifying or ruling out nodal disease and incorporating *POLE* and MMR status in this group will assist the treating physician in recommending the need for adjuvant and possibly de-escalation of therapy. This raises questions on whether the decision for SLN mapping should be based on pre-operative molecular classification, and perhaps omitted for patients with *p53*mut tumors who will benefit from adjuvant treatment regardless of the assigned stage of disease [79]. The differences in molecular classification within the same histologic subtype and the impact on prognosis should be incorporated into future surgical SLN studies aiming to optimize management for patients with EC.

## 7. Future Perspectives

Besides being a diagnostic tool to determine the stage and guide adjuvant therapy, the therapeutic value of SLN mapping remains unclear. Due to the widespread adoption of SLN mapping for staging in EC, it would be very challenging to perform a RCT to evaluate the oncologic benefit of SLN compared to systematic pelvic and para-aortic lymphadenectomy for high-grade EC due to the previously determined benefits to these patients with regard to morbidity. In addition, these studies would have to incorporate molecular characterization of high-grade ECs, stratify based on TCGA subgroups a priori, and carefully standardize the administration of the adjuvant therapy [78]. Several ongoing trials are currently assessing some of these undetermined controversies. ENDO-3 [ClinicalTrials.gov/NCT04073706] is a non-inferiority RCT comparing progression-free survival in patients undergoing SLN mapping compared to no lymph node assessment in clinical stage I EC of all histologic subtypes with standardized adjuvant therapy [82]. This study will also compare adverse events, rates of lymphedema, and health-related quality of life. This will be the first study to compare these two groups, and the results might provide additional insights to optimize the surgical management of these patients.

## 8. Conclusions

SLN mapping is an appropriate diagnostic surgical tool to identify occult nodal metastases in patients with EC. Its high sensitivity and low morbidity make it an acceptable technique for lymph node assessment as identifying positive nodes is important for determining adjuvant treatment. While there had been agreement on the feasibility of SLN mapping in low-grade EC, its performance in high-grade histology had not been clearly established. Recent data have been more convincing on the diagnostic accuracy of this method, even in high-grade histology. Nevertheless, consensus on the implementation of SLN mapping is far from being achieved, especially in the era of molecular classification and individualized medicine. We have yet to determine when and for whom SLN mapping will be of diagnostic and/or therapeutic value. Future research should focus on the survival benefit of SLN mapping versus no lymphadenectomy with the integration of molecular classifications.

## Data Availability

Not applicable.
